# Forensic Diagnosis of Sudden Cardiac Death Due to Thrombosis Secondary to Coronary Artery Ectasia: A Description of Two Cases

**DOI:** 10.7759/cureus.102324

**Published:** 2026-01-26

**Authors:** France Evain, Marco Roffi, Quentin Chatelain, Marouchka Gerth, Sara Sabatasso

**Affiliations:** 1 Forensic Pathology, University Center of Legal Medicine (CURML), Geneva, CHE; 2 Forensic Pathology, Geneva University Hospitals, Geneva, CHE; 3 Division of Cardiology, Geneva University Hospitals, Geneva, CHE; 4 Forensic Pathology, University Center of Legal Medicine (CURML), Geneva University Hospitals and University of Geneva, Geneva, CHE; 5 Forensic Pathology, Geneva University Hospitals and University of Geneva, Geneva, CHE; 6 Forensic Pathology, Geneva University Hospitals, Lausanne, CHE; 7 Faculty Unit of Anatomy and Morphology, University Center of Legal Medicine (CURML) CHUV (Lausanne University Hospital) and University of Lausanne, Lausanne, CHE

**Keywords:** coronary, ectasia, forensic, histology, pathology

## Abstract

Coronary artery ectasia (CAE), whether it is diffuse or localized, is an uncommon finding. It is sparsely documented, especially by post-mortem imaging and histology. In this article, we will describe two cases of sudden cardiac death secondary to coronary thrombosis in the context of CAE. The cause of death was myocardial infarction in both cases. Diagnosis was based on post-mortem computed tomography angiography, forensic autopsy, and histological examination, offering a rare and complete illustration of the disease.

## Introduction

Sudden cardiac death is one of the main causes of unexpected fatal events at forensic autopsies [1]. While etiology varies with age, coronary atherosclerosis complicated by myocardial ischemia/infarction is the most prevalent cause in adults older than 35 years of age [2]. Coronary artery aneurysm (CAA) and coronary artery ectasia (CAE) are uncommon findings at autopsy, first described by Morgagni in 1761 and by Bougon in 1812 [3,4]. They may define an abnormal focal (aneurysm) or diffuse dilatation (ectasia) of the vessel wall of one or more coronary arteries [5]. Compared to "classical" coronary artery disease, these pathologies concern the vessel diameter, which is abnormally enlarged. CAE/CAA are often associated with coronary atherosclerosis, suggesting a common pathological process [6]. Frequently asymptomatic, CAA and CAE may also manifest as stable angina pectoris or acute coronary syndrome secondary to thrombus formation with distal embolization or vessel occlusion [7,8]. We present two fatal cases of CAE complicated by thrombosis. Post-mortem computed tomography angiography (PMCTA) was performed prior to autopsy and oriented the diagnosis, which was confirmed by internal examination and histology. The combination of PMCTA and histology allowed a remarkable illustration of these rare pathologies.

## Case presentation

Formal authorization for anonymous publication of the following cases was delivered by the prosecutors in charge of the cases (patient informed consent not applicable).

Case 1

A 57-year-old man was found unconscious in his bed by his son. Emergency medical services were alerted, and the physician identified definite signs of death (rigor and livor mortis). The man was pronounced dead on site. According to medical reports, he was known for poorly controlled arterial hypertension (despite dual antihypertensive medication) and smoking. Because of the relatively young age and the presence of a third person, a medicolegal autopsy was ordered by the prosecutor.

**Figure 1:**
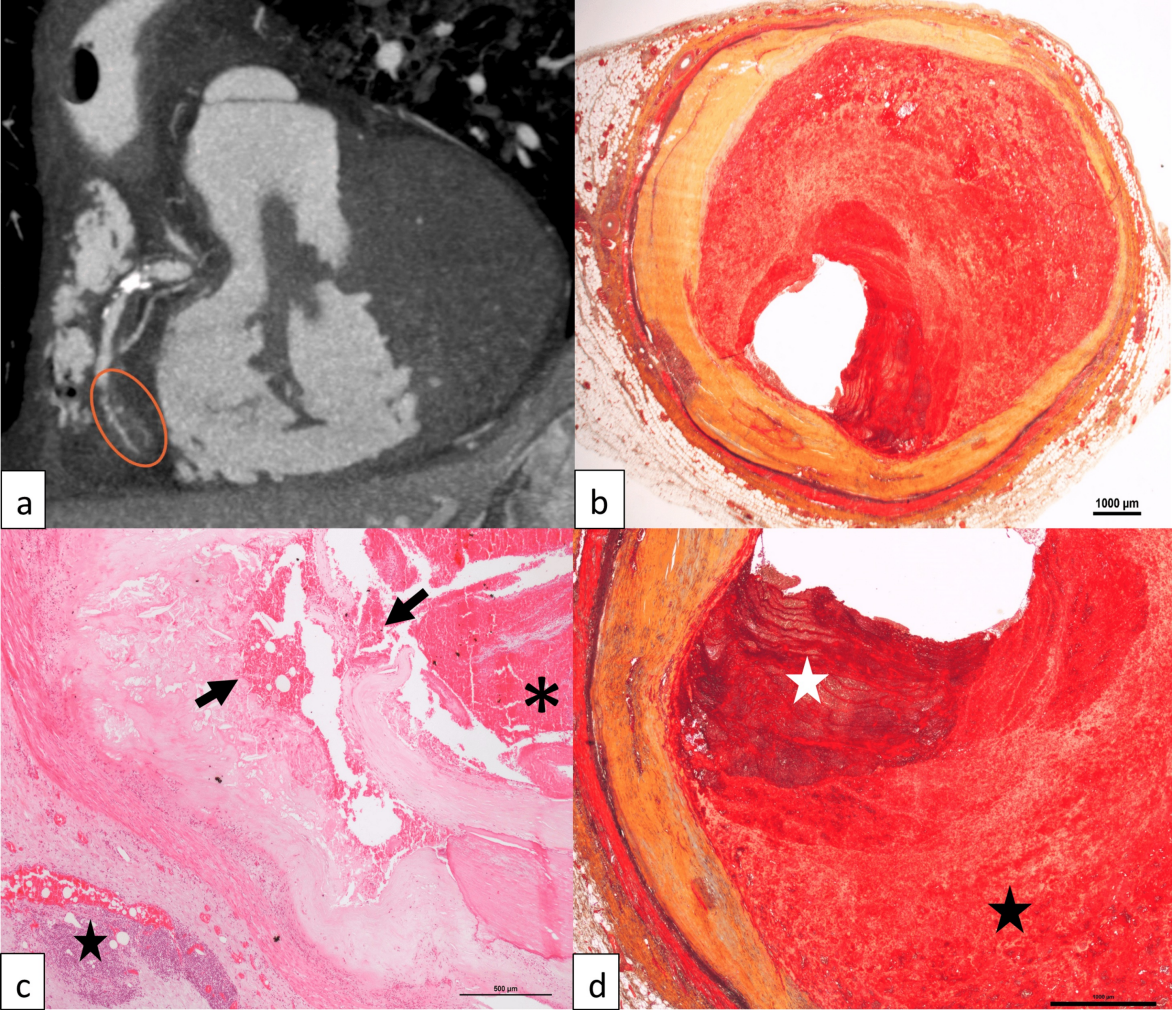
Case 1 (a) Post-mortem computed tomography angiography (PMCTA, arterial phase) showing a lack of opacification of the middle right coronary artery (orange ellipse). (b) Dilatation of the RCA (Movat pentachrome stain, 1x). (c) Plaque rupture and hemorrhage of the RCA (arrows), fresh thrombosis (asterisk), and chronic adventitial inflammation (star) (hematoxylin and eosin stain, 4x). (d) Old (black star) and fresh (white star) thrombosis of the middle RCA (Movat pentachrome stain, 2x).

Prior to autopsy, a PMCTA was performed and revealed diffuse coronary calcifications and a lack of opacification of the middle and distal segments of the right coronary artery (RCA) (Fig. 1a). Gross autopsy findings included cardiac hypertrophy (heart weight: 500 g), pulmonary edema and severe coronary atherosclerosis with a diffuse dilatation (ranging from 1 cm in the proximal left anterior descending coronary artery (LAD) to 1.1 cm in the mid RCA). A macroscopic stenosis of the proximal and middle segments of the RCA with thrombotic material was observed.

**Figure 2:**
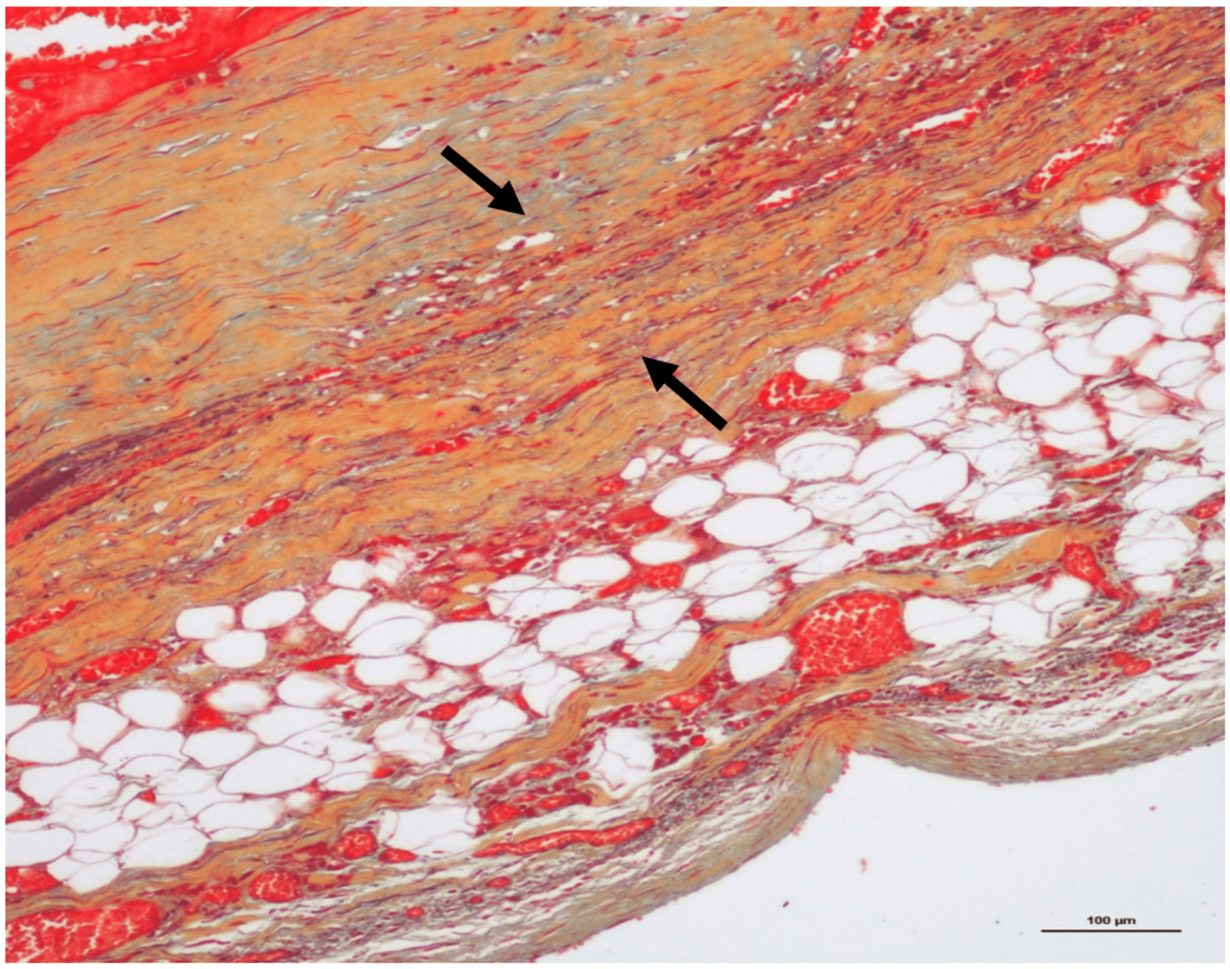
Case 1 Extreme thinning, almost disappearance (arrows), of the tunica media and elastic membranes of the RCA (Movat pentachrome stain, 10x).

**Figure 3:**
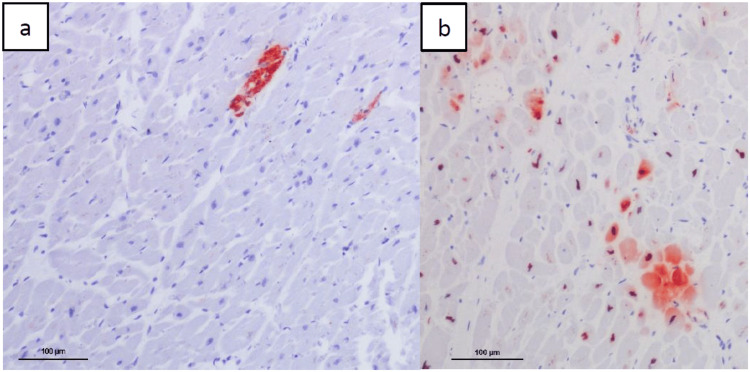
Case 1 Immunohistochemical examination of the lateral wall of the left ventricle showing small foci of myocardial necrosis. (a) Anti-C5b-9 antibody, 10x. (b) anti-fibronectin antibody, 10x.

Histological examination confirmed the coronary ectasia (Fig. 1b) and showed a ruptured plaque with an apposed fresh sub-occlusive thrombus in the proximal RCA, associated to an adventitial chronic inflammation (Fig. 1c). A fresh and ancient sub-occlusive thrombosis of the mid RCA was observed (Fig. 1d). Movat pentachrome and Miller trichrome stainings showed an extremely thin tunica media (sometimes not even visible), with an extreme thinning (almost disappearance) of internal and external elastic membranes (Fig. 2). Histological and immunohistochemical (i.e., anti-C5b-9 and anti-fibronectin antibodies) examination of the heart revealed signs of acute ischemia with small foci of myocardial necrosis (lateral and posterior walls of the left ventricle) (Fig. 3). Cause of death was an acute myocardial ischemia secondary to RCA thrombosis. Death was considered natural.

Case 2

**Figure 4:**
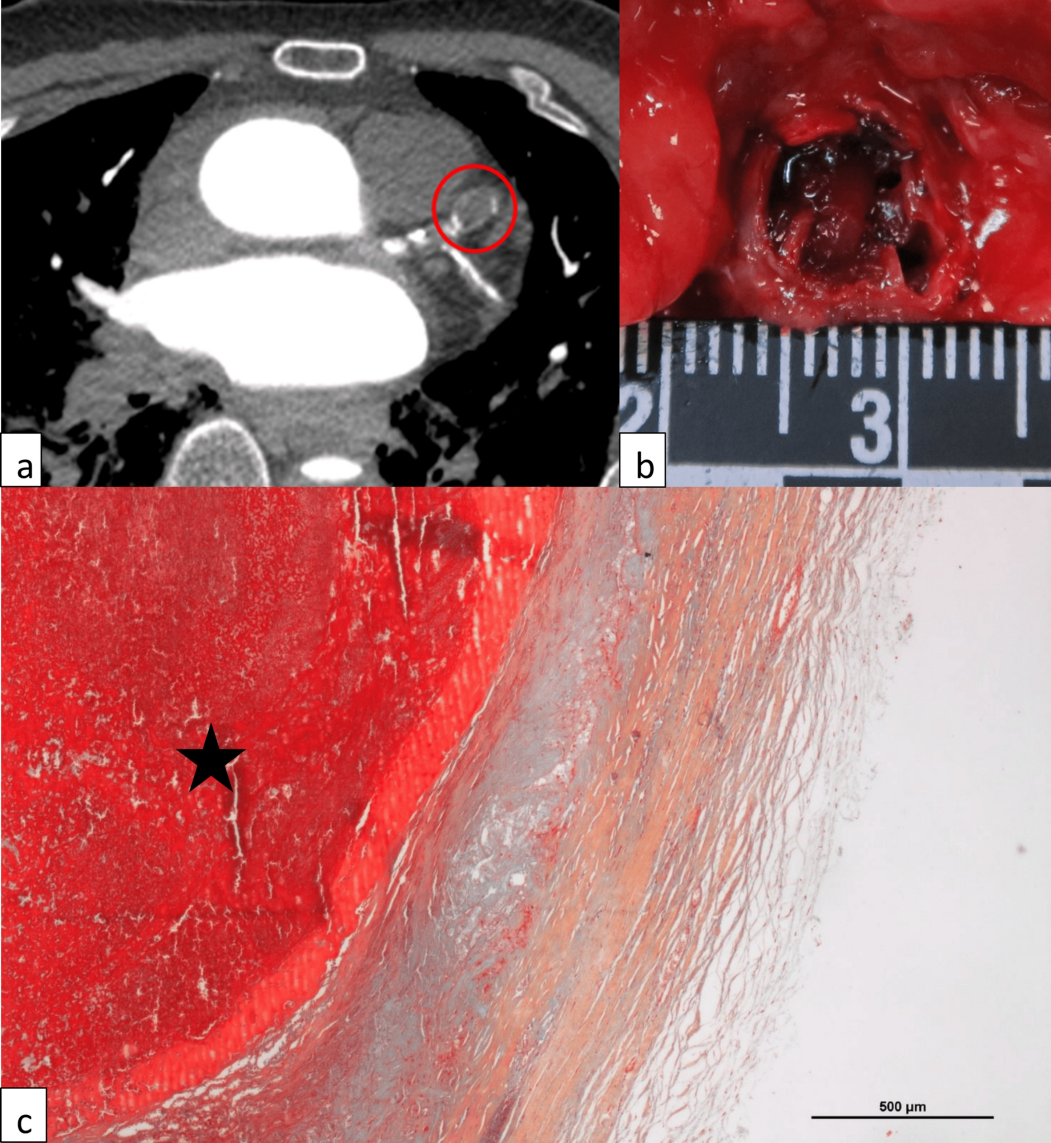
Case 2 (a) Post-mortem computed tomography angiography (PMCTA, arterial phase) showing a focal dilatation of the LAD (red circle). (b) Dilatation and thrombotic occlusion of the proximal LAD. (c) Disappearance of the tunica media and elastic membranes and fresh thrombosis of LAD (star) (Movat pentachrome stain, 4x).

A 66-year-old man known for arterial hypertension was found unconscious in the bathroom by his wife. Emergency medical service was alerted, and ventricular fibrillation was identified. The man was pronounced dead after 40 minutes of cardiopulmonary resuscitation. A medicolegal autopsy was performed the following day. PMCTA showed diffuse coronary calcifications and a lack of opacification of the entire length of the LAD and the proximal segment of the left circumflex coronary artery (LCX). Moreover, it revealed localized dilatation (aneurysms) of the proximal LAD (Fig. 4a) and other arteries (internal carotids, aorta, right common iliac). Macroscopic and microscopic examinations showed a chronic ischemic cardiomyopathy, an acute myocardial infarction of the posterolateral wall of the left ventricle, a severe coronary artery disease with occlusive to subocclusive stenosis (LAD, LCX, and RCA), a hypertrophic heart (900 g), and pulmonary edema. The localized dilatation of LAD was thrombosed, with fresh and ancient components (Fig.4b and 4c). Movat pentachrome and Miller trichrome stainings revealed a very thin (sometimes absent) tunica media of the coronary samples (Fig. 4c). The cause of death was myocardial infarction secondary to LAD thrombosis and associated subocclusions of the LCX and RCA. Death was considered natural.

## Discussion

In the literature, the denominations “coronary artery ectasia” and “coronary artery aneurysm” are used interchangeably, reflecting the lack of a uniform definition of this disease. CAE was defined by the Coronary Artery Surgery Study (CASS) as an abnormal diffuse dilatation of the vessel with a diameter 1.5 time greater than that of a healthy adjacent coronary artery [9,10], whereas an aneurysm may be restricted to a focal dilatation (localized ectasia) [5]. It is an uncommon finding at angiography in the Caucasian population, with a prevalence between 1.2% and 4.9% [6]. The pathology involves the proximal segments of the coronary arteries [9]. The RCA is affected in most cases (40-85.7%), followed by LAD (32-60%) and LCX (43-50.9%) [6,9,10]. Multiple coronary arteries can be affected simultaneously [5]. CAE mostly affects males (3:1), with a median age of approximately 60 years in clinical series [6,9]. Similar to aneurysms at multiple locations, risk factors include atherosclerosis, diabetes, hypercholesterolemia (including familial hypercholesterolemia), smoking, and cocaine use [6,11]. CAE can also be observed in connective tissue diseases (e.g., Ehler-Danlos, Marfan), vasculitides (e.g., Kawasaki, Takayasu), infections (mycotic), or following a coronary procedure (iatrogenic dissection, use of balloon or inadequately sized stent, atherectomy) [6].

The pathophysiology is not fully understood. As the two cases presented, most patients suffer from concomitant coronary atherosclerosis, suggesting a common pathophysiology [3]. A proposed mechanism is an exaggerated expansive remodeling of the vascular wall resulting in the dilatation of the lumen due to an enzymatic degradation of the tunica media secondary to the activation of matrix-degrading enzymes (matrix metalloproteinases) [3,5,6]. This could be triggered by several factors involved in atherosclerosis (accumulation of lipoproteins and foam cells formation, inflammation, renin-angiotensin system activation, and oxidative stress) [5,6]. At the same time, disturbance of the blood flow (turbulence, stagnation of blood) in the dilated coronary segment promotes thrombus formation, with a risk of distal embolization or vessel occlusion and myocardial ischemia [3,6,9]. The histological hallmark of CAE, as observed in our cases, is a degeneration of the musculo-elastic components of the vascular wall, notably the elastic membranes and tunica media [5,12].

CAE can be asymptomatic or manifest as exercise-induced angina pectoris (even in the absence of significant stenoses, due to slow flow), acute coronary syndrome, and myocardial infarction [3,13]. In the clinical setting, the gold standard for the diagnosis is coronary angiography. It provides information about the lumen, shape, and number of vascular dilatations, as well as prognostic information (severity and extension of the lesion, existence of concomitant stenoses) [3,12]. Intravascular ultrasound (IVUS) is the invasive imaging modality of choice to assess the size of the dilatation, vascular wall changes (differentiation between true and false aneurysm), and the presence of a coexistent stenosis [14]. Noninvasive imaging modalities (transthoracic echocardiography, CT angiography, and MRI) can also be used to detect CAE [3]. It should be noted that CT angiography allows the visualization not only of the coronary lumen, but also of the size and shape of the vascular dilatation, as well as of eventual thrombi (which may lead to an underestimation of the true vessel size on coronary angiography).

Management’s guidelines for CAE are lacking. Since atherosclerosis is the most common cause, statins are indicated. Antiplatelet agents are commonly proposed [6,7]. Vasodilators are not recommended without stenosis because of the risk of an even slower blood flow precipitating thrombus formation in the dilated segment [6,7,12]. An aggressive control of cardiovascular risk factors and implementation of lifestyle changes are mandatory. In case of ongoing myocardial ischemia/infarction and vessel occlusion, percutaneous intervention (PCI) with stenting is the treatment of choice, in association with thrombectomy if needed [3,7].

Long term prognosis of CAE is unknown. A review of the CASS (1983) stated that there is no survival difference between patients with CAE/CAA (n = 978) and patients with atherosclerotic coronary disease (n= 15’249) [15]. However, at that time, medical treatment of the latter was rudimentary and not comparable to today’s standards. In their study, Willner et al. stated that patients carrying both atherosclerotic coronary artery disease and CAE (n= 121) have a higher mortality rate and are more prone to complications compared to patients with isolated CAE (n = 40) [9].

## Conclusions

CAE (either diffuse or localized) is an uncommon pathology that can affect all coronary arteries and lead to thrombosis with acute myocardial ischemia/infarction, and sudden death. In the last scenario, these cases are referred to a forensic pathologist in order to exclude non-natural death. Pathophysiology is not fully understood and converges towards an excessive remodeling of the vascular wall. Histology is characterized by degeneration of the musculo-elastic components of the vascular wall, notably the elastic membranes and tunica media. Most CAEs are associated with coronary atherosclerosis. The prognosis is unknown, and the risk of associated (recurrent) myocardial infarction cannot be adequately estimated. Whenever possible, the treatment is conservative. Revascularization is reserved for advanced disease or ongoing ischemia. In this paper we presented two fatal cases of coronary artery thrombosis secondary to coronary ectasia, comprehensively documented by post-mortem imaging and histology, thus offering a rare and complete illustration of this pathology.
